# Diet of bird‐like troodontid dinosaurs: synthesis of a contentious clade

**DOI:** 10.1002/brv.70145

**Published:** 2026-02-13

**Authors:** Yui Chi Fan, Case Vincent Miller, Michael Pittman

**Affiliations:** ^1^ School of Life Sciences, The Chinese University of Hong Kong Shatin Hong Kong SAR China; ^2^ Section of Vertebrate Paleontology Carnegie Museum of Natural History 4400 Forbes Avenue Pittsburgh, Pennsylvania PA 15213 USA

**Keywords:** troodontids, diet, dietary evolution, dietary ecology, theropods, palaeobiology

## Abstract

Troodontidae is a clade of small‐to medium‐sized maniraptoran theropods that mainly lived in Laurasia (modern Asia, North America and Europe) during the Jurassic and Cretaceous periods and are believed to have had a variety of diets. The uniqueness of troodontid teeth suggests that they diverged from the typical flesh‐based diet of non‐avian theropods. Some Asian troodontids even lack tooth serrations completely, which has been linked to herbivory or omnivory. However, troodontids still possessed multiple traits suited for predation. These include a large curved second pedal digit; laterally compressed, curved, and serrated teeth; and ‘puncture‐and‐pull’ microwear on their teeth. Extrinsic evidence, such as stomach contents and gastric pellets, supports a diverse dietary intake among troodontids with evidence that they ingested both plant and animal material. Environmental, morphological, and biogeochemical analyses suggest that the iconic North American troodontid, *Troodon*, was likely to have been omnivorous, potentially preying on both plants and small animals, such as mammals and baby dinosaurs (hatched or unhatched). Our understanding of different diets among troodontids remains limited due to the sparsity of both relevant fossil material and palaeodiet research. Nevertheless, what information we have on troodontid diet informs ancestral deinonychosaurian and paravian diets, which we suggest were omnivorous. We encourage research into troodontids outside the Late Cretaceous of North America and further study of troodontid biogeochemistry and postcranial anatomy to improve our understanding of troodontid diet and the larger story of theropod ecological diversity.

## I. INTRODUCTION

### (1) Troodontidae

**Fig. 1:**
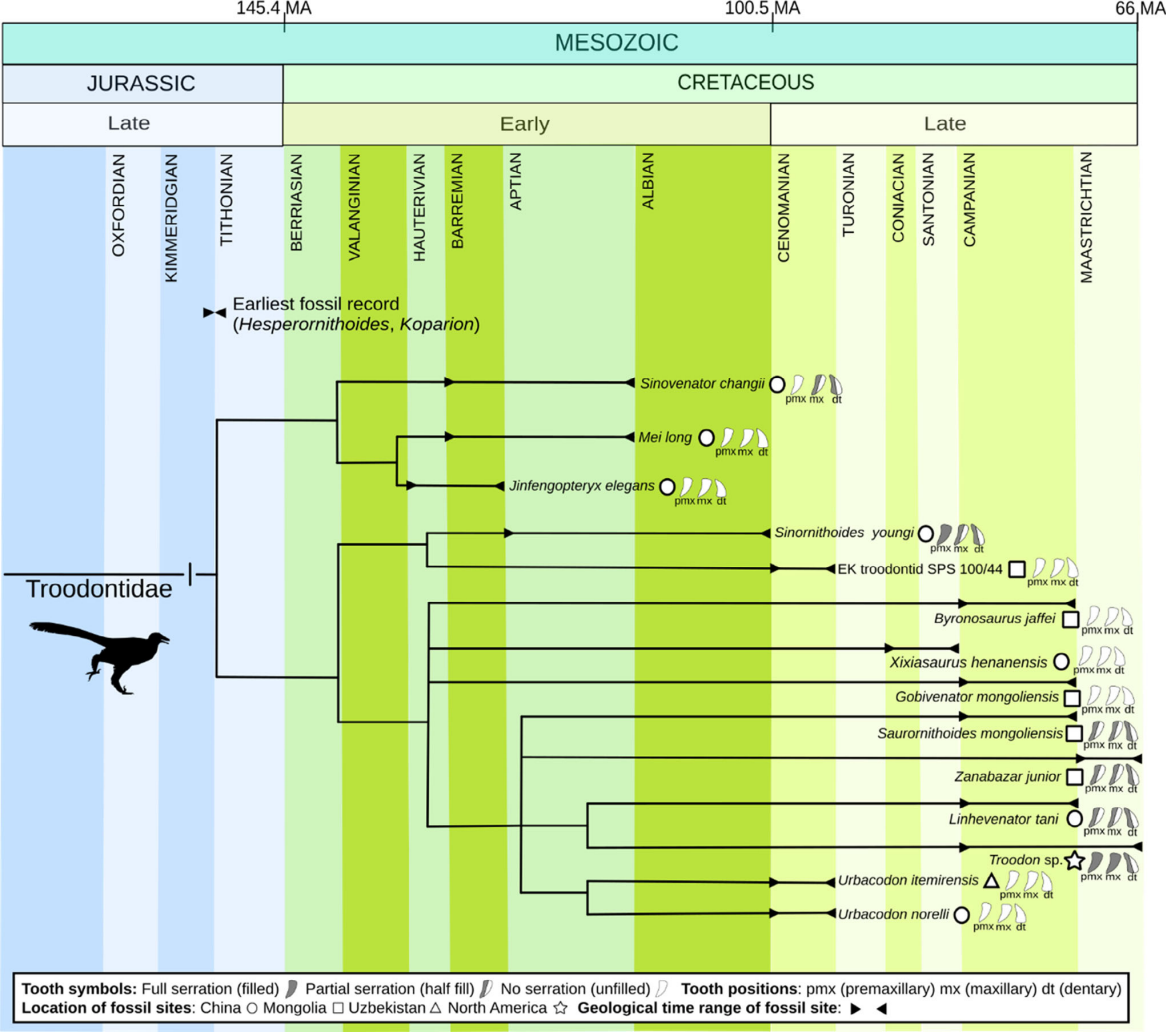
Simplified time‐calibrated phylogenetic tree of Troodontidae. Tooth symbols indicate serration positions for all teeth on a given tooth‐bearing bone. Dentary tooth serrations are the same as the corresponding premaxillary or maxillary teeth in all studied taxa, except *Troodon*, whose dentary teeth have serrations only on the distal carinae. All taxa except *Troodon* sp. are found in Asia. Tree topology modified from Fig. 1 of Pei *et al*. (2020) and Fig. 7 of Wang *et al*. (2024). MA, million years ago.

Troodontidae are a group of small, bird‐like theropods belonging to the clade Paraves. The clade is defined as a stem‐based monophyletic group containing *Troodon formosus* and all coelurosaurians closer to it than to *Velociraptor mongoliensis* or *Passer domesticus* (Turner, Makovicky & Norell, 2012) (Fig. 1). Whilst taxa traditionally thought of as troodontids are still recovered in the defined troodontid clade, some studies have recovered these taxa in alternative phylogenetic positions, although always still as close relatives of bird and dromaeosaurid taxa (discussed in Pittman *et al*., 2020).

### (2) Troodontid biogeography

**Fig. 2:**
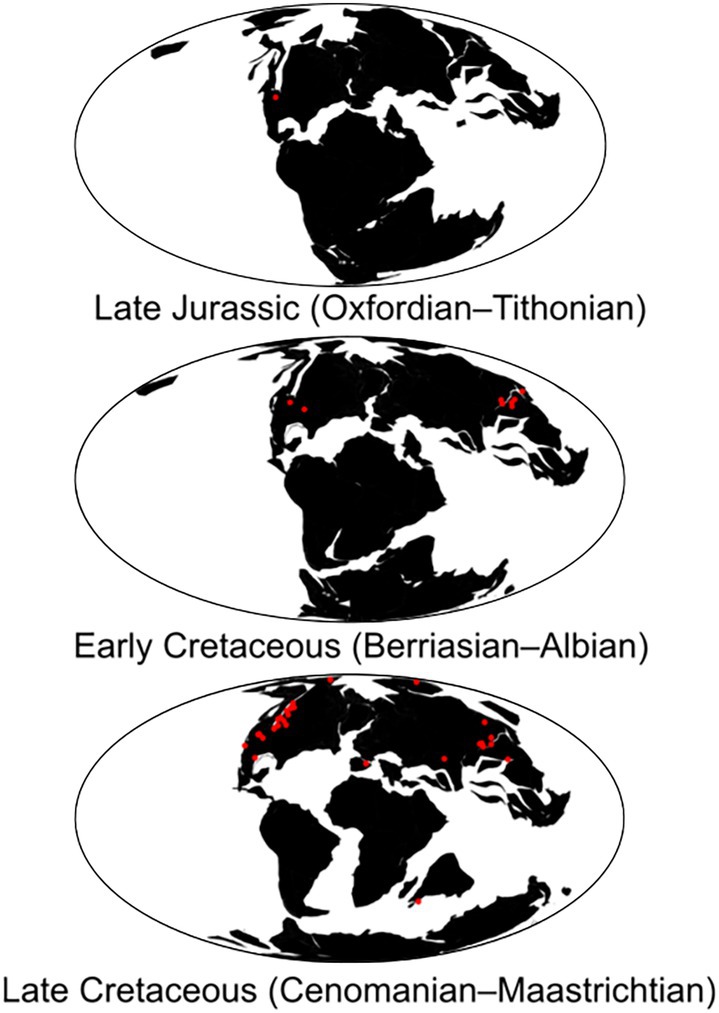
Global distribution of troodontid fossil sites (shown in red) overlaid onto palaeogeographic reconstructions of the Late Jurassic (Oxfordian–Tithonian), Early Cretaceous (Berriasian–Albian), and Late Cretaceous (Cenomanian–Maastrichtian). Maps modified from Paleobiology Database Navigator (https://paleobiodb.org/navigator/) (Müller *et al*., 2018). Troodontidae fossil distributions drawn from Ding *et al*. (2020) and Pittman *et al*. (2020).

The earliest troodontids date to the Late Jurassic of North America (Hartman *et al*., 2019; discussed in Pittman *et al*., 2020). Most troodontid specimens were discovered in Late Cretaceous formations of Campanian and Maastrichtian age in Asia and North America (van der Reest & Currie, 2017; Pittman *et al*., 2020), but Early Cretaceous troodontids are also well‐represented from Asia. Suspected troodontid fragments are reported from Late Cretaceous Europe as well (Vullo, Neraudeau & Lenglet, 2007; Sellés *et al*., 2021), which we agree should be attributed to Troodontidae (Fig. 2). Troodontids were traditionally thought of as a Laurasian clade, but a single tooth (DUGF/52) found in the Late Cretaceous of India potentially extends their record to Gondwana (Goswami *et al*., 2013; Pittman *et al*., 2020). However, this tooth specimen lacks diagnostic traits of later‐diverging troodontids (globose crown and hypertrophied denticles), and its troodontid origins have been questioned (Agnolin *et al*., 2019).

### (3) Validity of *Troodon*

*Troodon* is a key taxon in troodontid‐related research. Multiple studies use the genus as a proxy for Troodontidae as a whole, giving *Troodon* increased importance when considering troodontid dietary reconstruction. Many controversies surround the single non‐diagnostic holotype tooth of *Troodon*, especially taking into account that tooth morphology appears to be insufficient to distinguish *Troodon* specimens between different localities (Torices *et al*., 2014; Evans *et al*., 2017). Additional issues include a lack of certainty about the location of the holotype quarry and a lack of overlapping material to assign other named taxa to it (Cullen *et al*., 2022; Cullen & Cousens, 2024). This review supports the seniority and usage of *Troodon* as a proper genus name, based on naming conventions, the broad usage of *Troodon formosus*, and morphological examinations linking later, more complete fossils to the single‐tooth type specimen (Varricchio, Hogan & Gardner, 2025), although the proposed neotype is from a different geological formation (Two Medicine rather than Judith River Formation). Currently, the morphology of *Troodon* tooth specimens is found to be indistinguishable across different localities across North America (Torices *et al*., 2014; Evans *et al*., 2017). This can potentially support the wide dispersal of *Troodon* as a genus. However, given the existence of other troodontids in North America, the diversity of North American troodontids is probably significantly underestimated at present. Commonly accepted troodontid species from North America include *Stenonychosaurus inequalis* (Russell, 1969) (also recognised as *Troodon inequalis*; Currie & Koppelhus, 2005), *Latenivenatrix mcmasterae* (van der Reest & Currie, 2017), *Talos sampsoni* (Zanno *et al*., 2011) and *Hesperornithoides miessleri* (Hartman *et al*., 2019). However, the validity of *Stenonychosaurus inequalis* and *Latenivenatrix mcmasterae* as separate species from *Troodon formosus* remains disputed (Varricchio *et al*., 2025). Fundamental aspects like morphological variation of tooth position remain unaccounted for in North American troodontids (Hendrickx, Tschopp & Ezcurra, 2020), further complicating issues of identification *via* isolated teeth.

The notion that *Troodon formosus* is a *nomen dubium* is not followed here. However, given the diversity of Asian troodontids in both distribution and morphology, it is plausible that *Troodon* could be considered a wastebasket taxon. While supporting arguments for *Troodon formosus* as a valid species have been presented by Varricchio *et al*. (2025), no definitive decision has been made concerning its taxonomic nomenclature or validity. This is relevant to palaeodiet reconstruction: if *Troodon* is a wastebasket taxon of multiple species with different dietary needs, then multiple trends of dietary evidence should appear. These divergent trends in evidence may or may not be distinguishable from an omnivorous and monophyletic *Troodon*. Currently, almost all western North American Late Cretaceous troodontid specimens are referred to *Troodon formosus* (Varricchio *et al*., 2025). Pending a thorough taxonomic review of referred material, this review considers that *Troodon* is currently best understood as a broad designation for North American troodontids to accommodate different interpretations of existing fossil material.

### (4) Troodontids and birds

Understanding troodontid ecology is a key step to understanding the transition from non‐avialan theropods to birds (both crown‐group birds, Aves, and their extinct relatives in Avialae) (Pittman & Xu, 2020). Birds are essential to modern global ecosystems, occupying multiple diverse niches and thriving in various habitats on an extremely wide range of nutrient sources (Rico‐Guevara *et al*., 2019) (see Table 1). Outside of the crown group, early‐diverging birds are also believed to have had diverse diets and a rapid rate of ecological diversification (Miller *et al*., 2024). What remains more obscure is how Avialae achieved this ecological diversity. Among birds and near‐bird theropods (Paraves), troodontid ecology stands out as the most poorly understood (Zanno & Makovicky, 2011). On this basis, we present a consensus of what we currently know about the diet of troodontids and how this can help further our understanding of birds as well as paravian theropods more broadly.

**Table 1:** Descriptions of animal diets. A glossary of categories used in this review as a framework for dietary descriptions based on Miller & Pittman (2021).

Diet	Description	Source
Carnivory	Energy acquired primarily by consuming animal tissue.	Ullrey (2018)
Hypercarnivory	A particularly high percentage of energy is acquired by consuming animal tissue.	van Valkenburgh (1991)
Herbivory	Energy is acquired primarily by consuming plant tissue.	Karban & Agrawal (2002)
Omnivory	Consuming a variety of foods, with no one source providing the majority of energy.	Thompson *et al*. (2007)
Predatory	Consuming tissue of animals killed by the consumer.	Taylor (1984)
Scavenging	Consuming tissue of animals not killed by the consumer.	Turner *et al*. (2017)
Raptorial	Predation in which the pes plays a significant role in killing and/or restraining the prey.	Fowler *et al*. (2009)

## II. CONSUMED AND EXPELLED MATERIAL

Fossilised meals are a direct indicator of the material consumed by extinct animals, specifically the last meal of an organism before its death. Gut contents, despite their rarity, have been reported in troodontids. The troodontid *Jinfengopteryx elegans* is known from a single specimen (CAGS‐IG‐04‐0801) that preserves unusual stomach contents (Ji, 2005), which were regarded as evidence for herbivory (Zanno & Makovicky, 2011). Originally described as either eggs, developing ovarian follicles, seeds, or nuts (Ji, 2005), the reddish‐yellow oval structures suffer from poor preservation. Based on this single specimen, we have potential evidence of at least one troodontid consuming plant material during its life. As this would be the last meal of this single *Jinfengopteryx elegans* individual, it should only be viewed as a single data point in reconstructing diet. Preservation biases and the possibility of that meal being the very thing that killed the animal make it difficult to use this as evidence of everyday diet (Miller & Pittman, 2021).

Gastric pellets are regurgitated masses of indigestible material such as feathers and bones. Common in living birds, these represent material which has been mostly digested and thus avoids the lethality question of fossil gut contents. Fossilised forms of gastric pellets (regurgitalites) have been attributed to *Troodon formosus* at the Cretaceous Egg Mountain locality of the Two Medicine Formation (Freimuth *et al*., 2021). Two aggregates with mammalian bones (MOR 10912 & 10913) were identified as regurgitalites. Freimuth *et al*. (2021) interpret the absence of phosphatic ground mass in the aggregates as evidence that they are unlikely to be coprolites (see Gordon *et al*., 2020), and similarities in preserved bones and level of corrosion are interpreted as evidence that the two specimens were created by the same predator taxon (Freimuth *et al*., 2021). Troodontids were identified as the most probable source of these proposed gastric pellets based on a process of elimination against other predators at the locality, as well as behavioural (abundant nesting activity at locality) and phylogenetic (close relationship with living birds and the early bird *Anchiornis* (Zheng *et al*., 2018) which has known associated regurgitalites) evidence (Freimuth *et al*., 2021). While the size of these suspected pellets is consistent with small‐bodied theropod hunters, there could be other small‐sized tetrapods not yet described from the area. Furthermore, these materials were not directly observed near or within any fossilised organisms pointing to their origin (Freimuth *et al*., 2021). Thus, the troodontid origin of these suspected gastric pellets cannot be confirmed, but improved sampling of this locality could potentially increase the likelihood of the proposed association.

Based on fossil preservation of food remains and expelled material, there is dubious evidence for ingestion of both plant and animal material within Troodontidae. We consider the evidence of *Troodon formosus* as a small‐prey hunter (Freimuth *et al*., 2021) to be weaker. There is no association between the gastric pellets and *Troodon* body fossils, only hypothetical association based on outside factors. We consider this insufficient to determine dietary trends in troodontids. While plant‐based gut contents preserved in *Jinfengopteryx elegans* [assuming they are not ovarian follicles, which future testing (Mayr *et al*., 2020) may be able to resolve] cannot necessarily be used in isolation to reconstruct an herbivorous lifestyle, we can infer that Troodontidae had members that consumed plant materials to some extent.

## III. DENTAL MICROWEAR

Dental microwear is defined as the microscopic wear formed on the surface of a tooth as a result of usage (Ungar, 2015). Dental wear analysis is commonly applied to living and fossil mammals (Green & Croft, 2018), with recent application in theropods (Torices *et al*., 2018; Bestwick, Unwin & Purnell, 2019). Examining the abundance and orientation of wear scratches on teeth provides evidence of the chewing mechanism and physical properties of foods eaten. Teeth will scrape past food items when biting, leaving distinct wear marks in accordance with the biting motion and the substance being bitten. Torices *et al*. (2018) examined and compared microwear of 57 teeth from multiple theropod species, including tyrannosaurids, dromaeosaurids and the troodontid *Troodon inequalis*. Two types of scratch orientation were observed: parallel to subparallel scratches and oblique scratches. All theropod teeth had a group of scratches oriented parallel to the long axis of the tooth and a group oriented at an angle of ~30–40° to the carina. There were no noticeable differences in the microwear patterns between the large and small theropods sampled (Torices *et al*., 2018) indicating similar biting motions across the clades studied. All species have tooth wear that matched a ‘puncture‐and‐pull’ feeding motion seen in some living birds of prey (van Heteren *et al*., 2021) and predicted for non‐avian theropods (Erickson & Olson, 1996). This refers to the motion where the theropod bites into its prey before pulling dorsally to rip flesh and then swallowing. Scratches on *Troodon inequalis* teeth figured by Torices *et al*. (2018) appear longer, more numerous, and less parallel to the tooth margin than those in dromaeosaurid species (Miller & Pittman, 2021). This could potentially point to different dietary preferences in *Troodon* and contemporary theropods.

While scratches are commonly found, pits are rarely seen in troodontid specimens (Torices *et al*., 2018). Formation of microwear pits is correlated with biting bone or other hard materials (Hoffman, Fraser & Clementz, 2015). This suggests that *Troodon inequalis* did not regularly bite bone or other hard materials. Possible modes of carnivorous feeding include selectively removing flesh from bone or ingesting prey whole. In short, microwear analysis of *Troodon inequalis* teeth suggests a diet of relatively soft foods (Torices *et al*., 2018). This would most likely be animal tissue (Bestwick *et al*., 2019), although scratch orientation may suggest different predatory preferences than contemporary theropods.

## IV. ENVIRONMENTAL EVIDENCE

### (1) Shed teeth

Isolated teeth are common dinosaur fossils owing to the continuous replacement of dinosaur teeth. This, combined with the durability of enamel over geological timescales, contributes to a wealth of preserved theropod teeth (Smith, Vann & Dodson, 2005). Shed teeth provide vital environmental, biodiversity, and ecological information (Hendrickx *et al*., 2019), especially in places where body fossils are scarce. Troodontid teeth are no exception. Across North American formations, theropod teeth are attributed to *Troodon* due to their iconic large and hooked denticles. However, the uncertainty of diagnosing the genus, as discussed in Section I.3, creates difficulties when identifying this tooth material. In this context, we refer all troodontid teeth across different North American formations to *Troodon*, acknowledging that the true taxonomic diversity of North American Troodontidae was probably higher (Torices *et al*., 2014). Even when considering teeth across Theropoda as a whole, shed teeth remain difficult to assign to family and genus levels due to widespread homoplasy in their morphology (Torices *et al*., 2014; Hendrickx *et al*., 2020).

Despite difficulty in distinguishing genus‐level troodontid taxa in North American Upper Cretaceous formations, the locations of troodontid dental remains can provide clues for studying their diet. An unusually high abundance of *Troodon* teeth can be found in multiple North American fossil sites alongside newly hatched hadrosaurs (Whitebone, Funston & Currie, 2024). At Horseshoe Canyon Formation, troodontid shed teeth found in site L2000 represent over half of the isolated theropod teeth sampled (*N* = 65) and comprise about one‐third of identifiable dinosaur elements (Vickaryous *et al*., 1998). Troodontid shed teeth at site FTS‐2 (*N* = 29) are the most common theropod teeth and comprise a quarter of all dinosaurian teeth material found (Whitebone *et al*., 2024). All *Troodon* teeth from sites L2000 and FTS‐2 (except one from FTS‐2) are either broken below the crown or lack the root, suggesting that these were shed teeth. Baby hadrosaur elements were also found abundantly at the site, despite their remains being typically rare (Vickaryous *et al*., 1998; Whitebone *et al*., 2024). In the Horseshoe Canyon Formation, 18% of hadrosaur remains were babies with minimal tooth abrasion. Due to the fragility of baby‐sized hadrosaur teeth and bones, the minor abrasion on these remains further suggests that they were derived from a nearby source, such as a nesting site. The relatively unworn denticles indicate that they were not shed as old teeth but during feeding.

The co‐occurrence of troodontid shed teeth and baby hadrosaur elements has been noticed across North America (Vickaryous *et al*., 1998; Fanti & Miyashita, 2009; Druckenmiller *et al*., 2021; Whitebone *et al*., 2024). Combining the above lines of evidence, Vickaryous *et al*. (1998) suggested that troodontids may have been feeding, if not preferentially then at least opportunistically, on hadrosaurid eggs and/or babies at the site. We propose an alternative interpretation: baby hadrosaurs and *Troodon* may have shared a food source. A hadrosaur nesting site is likely selected for resources suitable for baby hadrosaurs, e.g. soft plants, which *Troodon* may have fed on as well. Both interpretations need not be mutually exclusive; *Troodon* may have been taking advantage of both easy‐to‐obtain food sources. Shed teeth results may also include cryptic species that face considerable difficulty in being further differentiated with current technology (Torices *et al*., 2014). This compounds difficulties in robustly interpreting shed teeth in fossil associations. In this case, the association could indicate carnivory, herbivory, or both in *Troodon*, and could even indicate multiple cryptic species taking different foods.

### (2) Digging traces

**Fig. 3:**
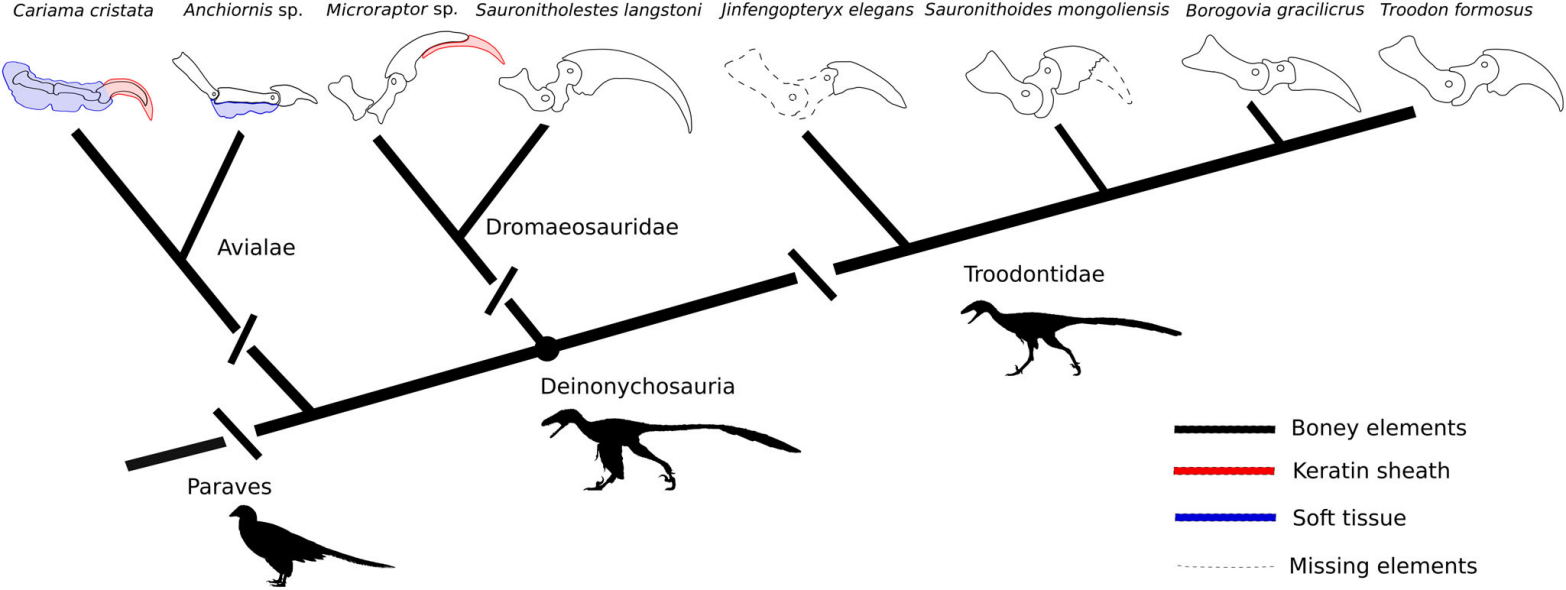
Second pedal digit of selected Paraves. Extant species, red‐legged seriema (*Cariama cristata*) sourced from Wildlife World Zoo, Aquarium, and Safari Park, Phoenix, AZ, USA (from Oswald *et al*., 2023). Extinct species: *Anchiornis* sp. (STM 0–147); *Microraptor* sp. (STM 5–109); *Sauronitholestes langstoni* [combined from RTMP 88.121.39 (11–1, 11–3) and 80.16.1318 (11–2, right side, image reversed)]; *Jinfengopteryx elegans* (CAGS‐IG‐04‐0801, from Ji, 2005); *Sauronithoides mongoliensis* (IVPP V10597); *Borogovia gracilicrus* from Osmólska (1987); *Troodon formosus* (CMN 1650; right side, image reversed). Drawings modified from Fig. 2 of Currie & Peng (1993) and Fig. 2 of Ji (2005).

Trace fossils record the interaction between an organism and the substratum (Hasiotis *et al*., 2007) and, importantly, record behaviours that may not be obvious from anatomy alone. Simpson *et al*. (2010) report a trace fossil association of maniraptoran digging traces and mammalian den complexes in the Wahweap Formation of the USA. The association is composed of three types of trace fossils: Type 1 traces are interpreted as holes dug by maniraptoran theropod dinosaurs. Types 2 and 3 are complex burrow systems identified as being produced by Mesozoic mammals (Simpson *et al*., 2010). The Type 1 trace makers were identified as probably ‘either dromaeosaurids or troodontids’ (Simpson *et al*., 2010, p. 699) owing to the general sparsity of information on how keratin sheaths change the shape of dinosaur claws, as well as the broad similarity between the ungual claws and keratin sheaths of dromaeosaurids and troodontids. However, as the Type 1 claw impression is highly recurved, but also short and robust, the maker is more likely a troodontid rather than a dromaeosaurid (Fig. 3). The association therefore suggests a troodontid (or deinonychosaurian) digging into the earth to hunt burrowing mammals. Mechanical tests partially support dromaeosaurids being able to dig prey out of burrows (Bishop, 2019), but no tests have been done on troodontids to assess this possibility. Digit II has been associated with predatory actions in paravians (Fowler *et al*., 2011; Pittman *et al*., 2022). Thus, predatory behaviour may also explain the enlarged second pedal digit of troodontids, although differences in this feature between troodontids and dromaeosaurids point to divergence in their feeding behaviour (see Section V.4 for further details).

## V. MORPHOLOGY

### (1) Tooth macrostructure

Tooth shape generally reflects an animal's diet. The shape of the teeth influences how they break down food during biting (Button & Zanno, 2020), and teeth are shaped in turn by contact with ingested material and occluding with other teeth during feeding processes (Green & Croft, 2018). Carnivores typically exhibit recurved teeth and a vertical dentition, in order to grip the flesh of struggling prey (Presch, 1974) and to transmit forces better during jaw adduction (Button, Rayfield & Barrett, 2014). In theropods, carnivorous teeth tend to exhibit the ‘ziphodont’ morphotype: laterally compressed, curved, and serrated (i.e. denticulated) (Prasad & de Broin, 2002). Herbivores and omnivores, by contrast, display a wider array of tooth morphologies. Tooth morphologies associated with herbivory include: procumbent dentition (Sues, 2000); conical dentition (Zanno & Makovicky, 2011); mesiodistally expanded, labiolingually compressed teeth with coarse denticles (Throckmorton, 1976); and loss of denticles and/or miniaturisation in tooth size (Weishampel, Dodson & Osmólska, 2004). Conical and coarse‐denticled dentitions are those most associated with herbivorous theropods (Zanno & Makovicky, 2011; Button & Zanno, 2020).

**Fig. 4:**
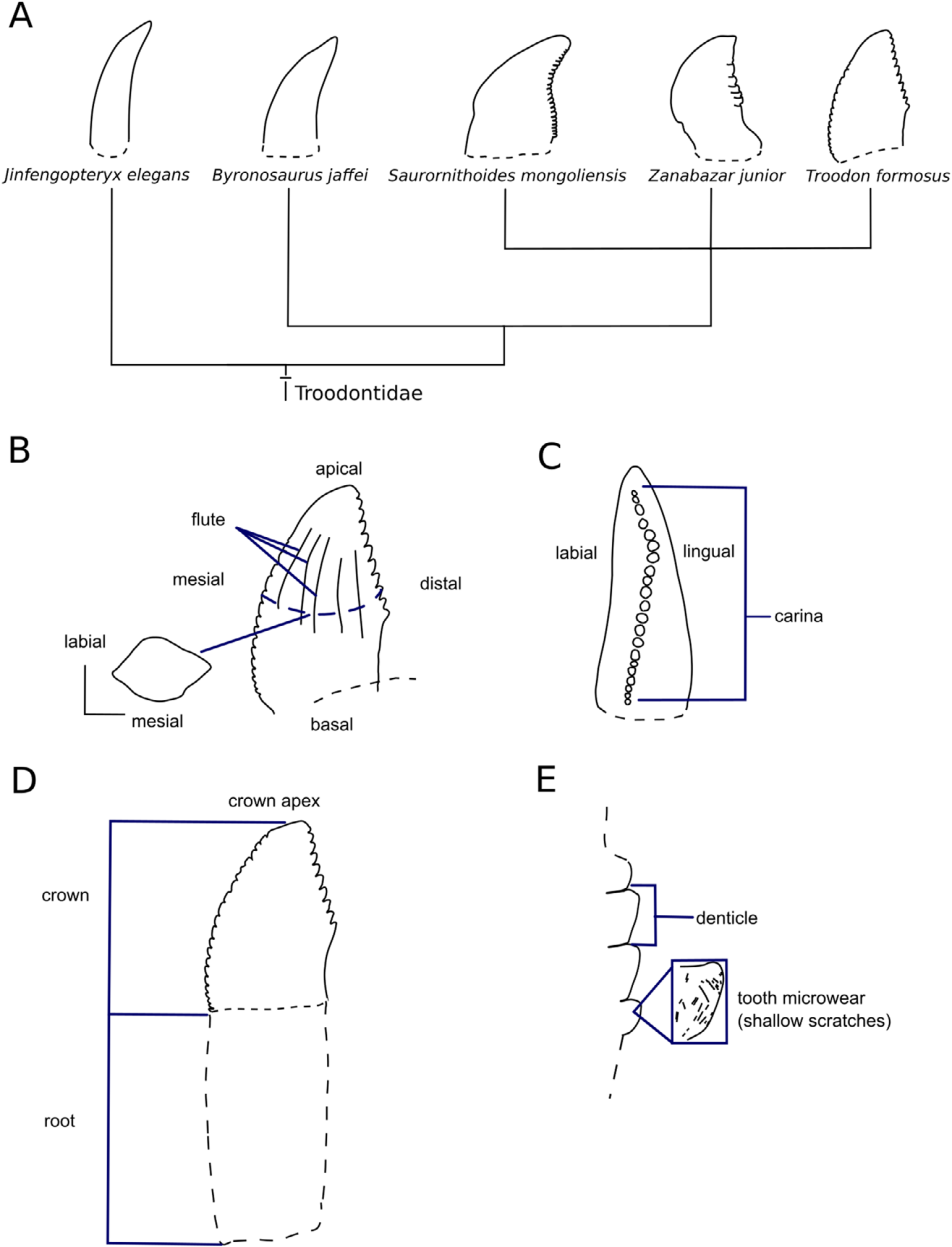
Troodontid tooth morphology. (A) Morphological diversity exemplified by *Jinfengopteryx elegans* (CAGS‐IG‐04‐0801), *Byronosaurus jaffei* (IGM 100/984), *Sauronithoides mongoliensis* (AMNH FR 6516), *Zanabazar junior* (IGM 100/1) and *Troodon formosus* (UVALP 55489). *Jinfengopteryx elegans* and *Byronosaurus jaffei* have teeth without denticles and serrations. The denticles on the teeth of *Troodon formosus* and *Zanabazar junior* are relatively large, whereas those on *Sinornithoides mongoliensis* are smaller and more densely packed. (B–D) Troodontid tooth crown anatomy, based on *Troodon formosus* tooth specimen UVALP 55489. (B) Labial view of a typical *Troodon* sp. tooth, including mid cross‐section view. (C) Labial view of *Troodon* sp. tooth where denticles form the carinae. (D) Labial view of tooth crown, including tooth root represented by dotted lines. (E) Close up of the coarse and large denticles of *Troodon formosus* (UVALP 55489). Microwear patterns modified from *Troodon inequalis* (UALVP 55303) from Torices *et al*. (2018).

Troodontids with serrated teeth, including *Sinovenator changii*, *Saurornithoides mongoliensis*, *Zanabazar junior*, *Troodon formosus* and *Linhevenator tani*, have tooth morphology distinct from other theropods (Currie, 1987; Farlow *et al*., 1991; Holtz, Brinkman & Chandler, 1998; Xu *et al*., 2011) (Fig. 4). Their tooth morphology has been interpreted as indicating both herbivory (Holtz *et al*., 1998; Zanno & Makovicky, 2011) and carnivory (Currie & Dong, 2001; Hendrickx *et al*., 2019). They possess serrated teeth like most theropods, yet differ in having larger denticles (Barsbold, 1974; Currie, 1987) (Fig. 4E). These coarse denticles are reminiscent of herbivorous dinosaurs (Holtz *et al*., 1998; Zanno & Makovicky, 2011) such as sauropodomorphs (Barrett, 2000), ceratopsians (Tanoue, You & Dodson, 2009), ornithopods (Strickson *et al*., 2016) and herbivorous therizinosaurian theropods (Zanno, 2010). Pachycephalosaurid teeth have been noted to be particularly similar to those of *Troodon formosus* (Gilmore, 1924). Pachycephalosaurid premaxillary teeth (UALVP 2) share similar curved dentition and small, packed serrations along the carina with troodontid maxillary teeth (e.g. *Troodon*, UAVLP 55376) (Sues & Galton, 1987). Troodontids also share a restriction at the base of the tooth crown (Hendrickx *et al*., 2019) with small‐sized ornithischians (thescelosaurids and pachycephalosaurids) (Sues & Galton, 1987; Boyd, 2014). Similarities in tooth shape in troodontids and small ornithischians may suggest similarities in diet.

However, Currie & Dong (2001) note several traits that unite troodontids with hypercarnivorous theropods: individual denticles curving distally towards the tip of the tooth, sharp enamel ridges between adjacent denticles, and blood grooves at the base of teeth. Troodontids also lack the crown‐shaped maxillary and dentary teeth found in pachycephalosaurids and thescelosaurids. Given these contrasting signals, omnivory is the most widely predicted diet based on fossil teeth (Holtz *et al*., 1998; Larson, Brown & Evans, 2016; Torices *et al*., 2018; Li *et al*., 2020).

The distinct *Troodon*‐like dentition (curved and apicobasally short tooth crowns with constricted bases and large, hooked denticles) is not present in all members of Troodontidae. Denticle size appears to have gradually increased within the family over evolutionary time, although this trend is complicated by loss of serrations in certain members (Fig. 1). Several troodontids (e.g. *Mei long*, ‘EK troodontid’ IGM 100/44, *Xixiasaurus henanensis*, *Urbacodon itemirensis*, *Urbacodon norelli* and *Jinfengopteryx elegans*) lack serrations entirely. Others lost serrations only on a single carina (Hendrickx *et al*., 2019) or only in the mesial teeth (Hendrickx *et al*., 2019; Wang *et al*., 2024). This serration loss occurs in multiple lineages at different times and geographic locations (Bever & Norell, 2009; Lü *et al*., 2010; Wang *et al*., 2024) (Fig. 1), implying repeated selective pressure towards this state. One proposed reason for denticle loss in some troodontids is a dietary shift. Since unserrated teeth would appear to have lost their ability to slice meat (Lü *et al*., 2010), dietary divergence in Troodontidae may be reflected in different tooth use (Farlow *et al*., 1991). For example, Komodo dragons (*Varanus komodoensis*) exhibit different dentition in the juvenile stage and the adult stage: juveniles have smaller, reduced and/or absent denticles at the tooth crown apex and base, whereas adult teeth have larger and more numerous serrations (Maho & Reisz, 2024). Juveniles live arboreally and mainly feed on insects, lizards, and, to a lesser extent, rodents (Losos & Greene, 1988; Purwandana *et al*., 2016), whereas adults feed by removing flesh sections from large mammal prey without swallowing the entire prey at once (Purwandana *et al*., 2016; Maho & Reisz, 2024). These differences in diet and feeding strategy are proposed to be linked to the development of denticles (Maho & Reisz, 2024). In the same way, troodontids with unserrated carinae may have preferentially taken smaller prey and swallowed them whole (Brink *et al*., 2016; Torices *et al*., 2018), while those with serrations would be more able to disassemble larger prey (Brink *et al*., 2016; Torices *et al*., 2018). The early‐diverging troodontid *Jinfengopteryx elegans* (CAGS‐IG‐04‐0801) is noted to preserve suspected plant material in the gut region of the specimen (Ji, 2005), suggesting a possible connection between a lack of denticles and herbivory (Holtz *et al*., 1998).

### (2) Tooth microstructure

Microscopic features of dentine in paravian teeth provide additional clues to feeding ecology. Li *et al*. (2020) noted the presence of interglobular dentine in troodontid specimen PWM 5400400036 (assigned to *Troodon* by the authors) in the porous interglobular space (IGS) between the dentine and enamel. The IGS is believed to play a key role in the redistribution of stress within a tooth to prevent breakage (Wang *et al*., 2015), and Li *et al*. (2020) did note that cracks in troodontid teeth stop at the IGS. Interestingly, they also noted that only teeth with serrations have cracks that reached the dentine. Loss of the IGS and thinning of enamel in avialans relative to *Troodon* and other non‐avian paravian theropods are proposed as reductions in tooth strength coincident with a dietary shift away from hard foods (Li *et al*., 2020). Analysis of tooth dentine tubule density shows that *Troodon* was not well suited to processing tough material, being closer to the condition of hypercarnivores than to strict herbivores (Brink *et al*., 2016). However, Li *et al*. (2020) noted that troodontids have higher tubule density (thus higher tooth strength) than avialans. Taken together, these results suggest that *Troodon*'s teeth were stronger than those of avialan theropods, but lack adaptations seen in obligate herbivores.

The observations of Li *et al*. (2020) may serve as an explanation for serration loss in troodontids. In the paravian teeth they studied, they found that cracks in the enamel only reach the dentine in serrated tooth specimens, while unserrated teeth have shallower cracks that remain within the enamel. This makes logical sense, as serrations increase the effective coefficient of friction between tooth and substrate and load the tooth with a higher percentage of the biting force (Frazzetta, 1988). Large‐denticled troodontid teeth are known to have a high turnover rate (Vickaryous *et al*., 1998), likely due to high rates of crack propagation through the tooth. So, the loss of denticles may allow the teeth to crack less, last longer, and reduce overall turnover rate. Growing fewer new teeth means lower resource requirements for the animal, providing a potential evolutionary advantage. To our knowledge, this mechanism of serration loss in troodontids has not been proposed before.

### (3) Cranium and mandible

The jaw of most animals forms a third‐class lever during a bite: the jaw joint acts as the fulcrum; the object being bitten acts as the load; the effort is the muscle (adductor muscle) that links the lower jaw to the cranium (Davis *et al*., 2010). Given the challenges that various food items present for ingestion, it is natural that different diets select for different jaw morphologies.

Troodontid jaw morphology is generally consistent with carnivory. Troodontids lack the hooked snout of some early saurodomorphs (Barrett, 2000; Button, Barrett & Rayfield, 2016) and the bills of ceratopsians (Ostrom, 1966) or ornithomimosaurs (Norell, Makovicky & Currie, 2001; Barrett, 2005), both of which are indicators of herbivory (Zanno & Makovicky, 2011). Troodontids also lack deeper mandibles (Ballell *et al*., 2024) and skulls (Metzger & Herrel, 2005), and a relatively higher bite force (Aerts, De Vree & Herrel, 1997; Saulnier Masson *et al*., 2023), which are features associated with herbivory in modern squamates. Yet, troodontids share an upwardly sloped jaw shape (Yun, 2025) with some ornithischians. The slope is formed by the predentary bone in ornithischians (as seen in thescelosaurid NCSM 15728) (Boyd, 2014) and a more diagonally orientated mandibular symphysis in troodontids (Yun, 2025). The diagonal shape of the distal jaw in both groups would support greater torsional stresses (Nabavizadeh, 2016; Yun, 2025).

**Fig. 5:**
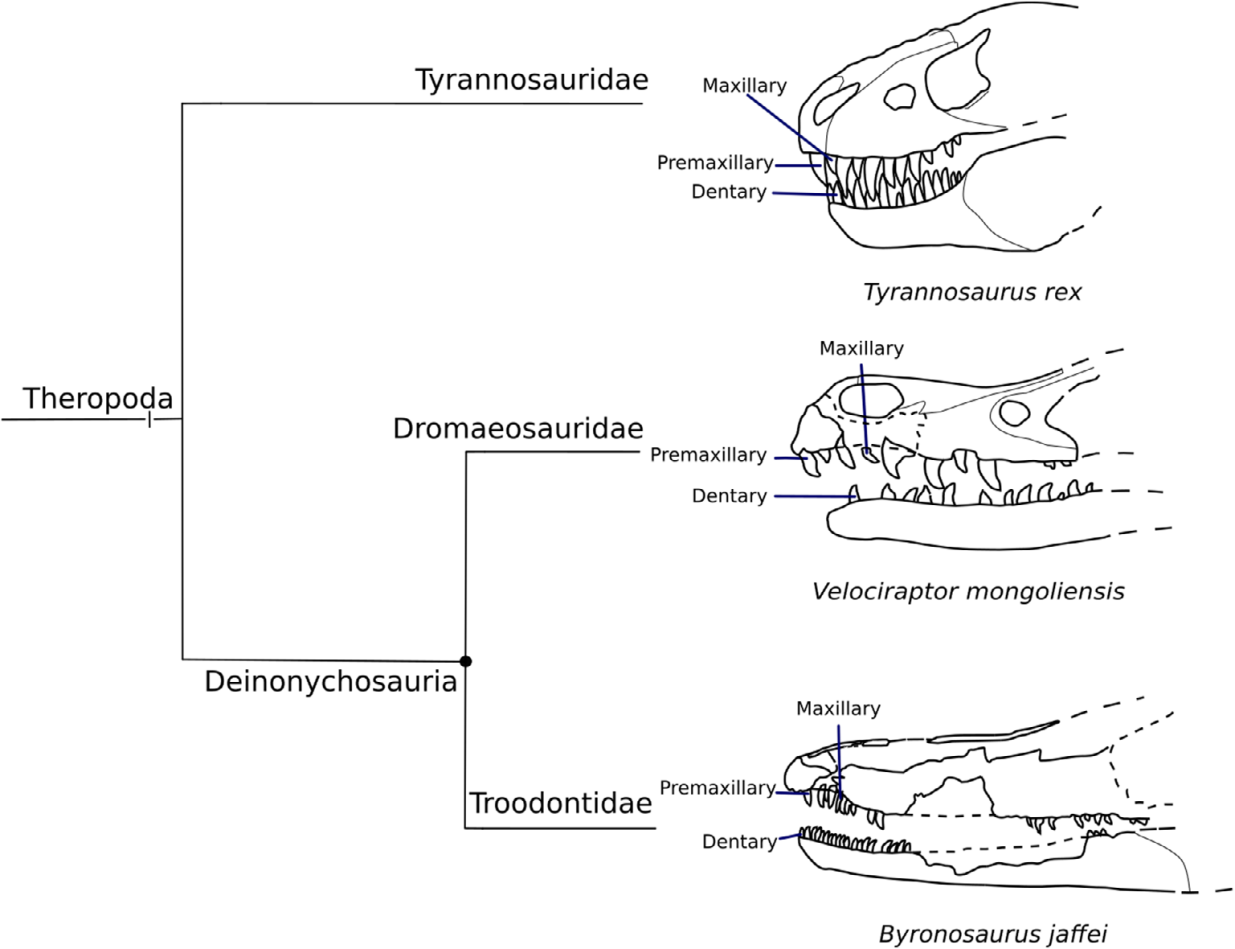
Skull shape and tooth type distribution in Tyrannosauridae and Deinonychosauria (Dromaeosauridae and Troodontidae). Tyrannosaurids show the most robust facial configuration out of the three skulls. By contrast, later‐diverging troodontids (*Byronosaurus jaffei*) have a less robust cranial profile, especially when compared with dromaeosaurids. *Byronosaurus jaffei* teeth are more numerous, smaller in size, and tightly packed. Drawings are of *Tyrannosaurus rex* (AMNH 5027), *Velociraptor mongoliensis* (AMNH FR 6515) and *Byronosaurus jaffei* (IGM 100/984).

Troodontids have relatively loose mandibular symphyses and movable intramandibular joints, reminiscent of predatory theropods and lizards (Holtz *et al*., 1998). However, troodontid jaws lack some predatory adaptations seen in contemporary theropods. Later‐diverging dromaeosaurids and many tyrannosaurids have a small and dorsally displaced maxillary fenestra (Fig. 5) which increases the structural strength of their skull (Pei & Xu, 2022). By contrast, late‐diverging troodontids (e.g. *Zanabazar*, *Saurornithoides* and *Gobivenator*) have a significantly elongated and anteroposteriorly positioned maxillary fenestra which lowers bite force resistance (Pei & Xu, 2022). The early‐diverging theropod *Coelophysis*, which is hypothesised to feed on small prey (Nesbitt *et al*., 2006), clusters with these later‐diverging troodontids in a geometric morphometric analysis of skull morphology (Pei & Xu, 2022). Taken together, skulls of later‐diverging troodontids share more similarities with theropods believed to specialise in small prey than those taking large prey (Fig. 5).

Troodontid jaw morphology creates a unique strength profile. Calculations of jaw strength for several troodontids (*Byronosaurus jaffei* MPC‐D 100/983, *Latenivenatrix mcmasterae* TMP 92.36.575, ‘*Polyodontosaurus grandis*’ CMN 8540, *Urbacodon itemirensis* ZIN PH 944/16 and *Zanabazar junior* MPC‐D 100/1) *via* beam theory show adaptation to endure mediolateral and torsional loads at anterior regions, interpreted as aiding in tearing plant matter, disassembling animals restrained by the pes, or shaking the head of their prey to kill them (Yun, 2025). The latter hypothesis may be explored through postcranial material as analogous behaviour in shrike birds requires rapid axial movements of the head (Sustaita, Rubega & Farabaugh, 2018) which likely requires distinct spinal adaptations. The anterior dentary expanded through the course of troodontid evolution (Yun, 2025), suggesting that whatever specialised function(s) it served in troodontids was under positive selective pressure. Tse, Miller & Pittman (2024) noted that the rostral shape of *Gobivenator mongoliensis* was likely ancestral to Deinonychosauria, suggesting the common ancestor of Deinonychosauria shared adaptations for rostral manipulation seen in early troodontids. The split between Troodontidae and Dromaeosauridae could represent the former group further specialising in its ancestral niche and the latter infiltrating different niches. For instance, later‐diverging dromaeosaurids have jaws primarily resistant to dorsoventral forces, which may have aided in rapid biting (Therrien, Henderson & Ruff, 2005; Therrien *et al*., 2021), saw‐motion biting (Sakamoto, 2010), and/or in disassembly of scavenged prey (Tse *et al*., 2024). We can thus consider these behaviours less likely in troodontids. While troodontid jaws do resemble tyrannosaurid jaws in their resistance to torsional loads (Therrien *et al*., 2021), we agree with the evaluation of Yun (2025) that this likely does not represent ecological convergence due to the large differences in the body plans of these clades.

### (4) Hind limb

Birds provide the strongest modern proxy for claw usage in extinct members of Paraves. In modern birds, the hind limbs are extensively used for feeding activities alongside the beak. In birds of prey, predatory actions involving the hind limbs include: delivering the initial strike (Goslow, 1971); restriction of prey by containment within the foot (Harris, 1984; Csermely & Gaibani, 1998) or pinning down under body weight (Fowler *et al*., 2011) depending on prey size; constriction by squeezing prey (Csermely & Gaibani, 1998); and piercing vital organs during restriction (Bond, 1942; Fowler, Freedman & Scannella, 2009).

Ungual morphology suggests troodontids were less adapted for grasping prey than dromaeosaurids. Both troodontids and dromaeosaurids possess a specialised second pedal digit (Osmólska, 1987). While this specialised digit may plausibly have served as a tool for digging or targeting vital areas of prey (Bishop, 2019), current consensus of the literature points to grasping and restraint as its most likely use (Fowler *et al*., 2011; Bishop, 2019). The second digit claw is larger and more strongly curved than the claws of other digits, and the articulations of the phalanges allowed for hyperextension (Bishop, 2019) (Fig. 3). The second digit claw is larger and more strongly recurved in dromaeosaurids overall, but troodontid unguals generally become larger and more recurved over evolutionary time (Xu *et al*., 2011; Hendrickx, Hartman & Mateus, 2015). Xu *et al*. (2011) suggests this indicates convergent use of the pes for hunting prey in both groups. However, troodontid unguals are less hinged (i.e. ginglymoid) than in dromaeosaurids (Fowler *et al*., 2011) and troodontids have an elongate third digit (Gianechini, Ercoli & Díaz‐Martínez, 2020), both of which resemble extant cursorial birds more than birds that kill prey in their claws. The modern red‐legged seriema (*Cariama cristata*), a largely cursorial bird that also possesses a hypertrophied second digit claw, likely presents the best analogy for troodontids. Oswald *et al*. (2023) observed captive red‐legged seriemas and found they could use their second digit claw to pin objects or prey in place during manipulation or disassembly with the beak. While high‐force grasping and killing of prey with the pes may have been possible for dromaeosaurids (Fowler *et al*., 2011), we consider seriema‐like low‐force pinning behaviour more likely in troodontids. While this naturally lends itself to a carnivorous interpretation of the group, it is not uncommon for living birds to pin or grasp plants and hold them in place during feeding (Clark, 1973).

Metatarsal morphology similarly suggests a more cursorial lifestyle for troodontids as well. Troodontids are known to have longer metatarsi than dromaeosaurids (Ascari, 2021) and exhibit an arctometatarsalian condition (Makovicky & Norell, 2004), which dromaeosaurids lack (Holtz, 1995). In extant raptorial birds, the metatarsus experiences a trade‐off: greater grip force with a shorter and robust metatarsus, or more rapid movements with a longer and more gracile metatarsus (Goslow, 1972; Ward, Weigl & Conroy, 2002). Fowler *et al*. (2011) suggested that the plesiomorphic cursorial metatarsus became further adapted towards cursoriality in troodontids, whereas dromaeosaurids specialised towards grasping strength at the expense of speed. Cursoriality does not have any single driver or ecological benefit (Carrano, 1999), so these findings serve more to rule out dromaeosaurid‐like predation than to suggest a particular dietary niche. Additional studies of extant birds with analogous hind limb structures to troodontids (e.g. Oswald *et al*., 2023) may shed additional light on this matter.

## VI. FINITE ELEMENT ANALYSIS

Finite element analysis (FEA) reconstructs stress, strain, and deformation in a digitised structure (Rayfield, 2007). FEA models help evaluate the relative performances of organism structures under a given load condition, allowing one to evaluate whether a structure is well adapted for a given role (Dumont, Grosse & Slater, 2009).

FEA has been carried out on North American troodontid teeth (referred to as ‘*Troodon*’ in this paper) (Torices *et al*., 2018). ‘*Troodon’* denticles had higher stress levels than the dromaeosaurids *Dromaeosaurus albertensis* and *Saurornitholestes langstoni*, especially at non‐optimal load angles (which simulate the angle at which teeth contact food). According to these models, ‘*Troodon’* would have been at greater risk of their teeth breaking at non‐optimal bite angles (Torices *et al*., 2018), such as biting into struggling prey. Food sources where bite angle could be precisely controlled, such as plants or carrion, may have been favoured. Notably, it is unclear whether the modelled ‘*Troodon’* tooth was a mesial or lateral tooth. According to the Morphosource data associated with the article (Torices *et al*., 2018), the specimen is an ‘isolated tooth’ and this information is likely unknown. Given the strength profile of the troodontid jaw (Yun, 2025), we may expect mesial teeth to be stronger than lateral teeth in troodontids. Models based on a specimen with *in situ* teeth (e.g. Norell *et al*., 2009) could test this hypothesis and potentially affirm or refute predictions of herbivory and scavenging.

FEA has not been performed on any other element of a troodontid, although work on the ungual claws of the dromaeosaurid *Velociraptor mongoliensis* (Manning *et al*., 2009) may be informative. The analysis indicated that the maximum stress experienced by the second ungual claw, under the assumed loading conditions of 400 N to be around 60 MPa, below the claw's predicted failure stress (Manning *et al*., 2009). Manning *et al*. (2009) interpret these data as *Velociraptor mongoliensis* being able to pull itself up with its claws. Unfortunately, biological FEA can only accurately reproduce the patterns of stress in a structure, not absolute values (Bright, 2014). So, these results present no valid ecological interpretations. Future studies may find success by either simulating multiple behaviours with different load conditions (Lautenschlager, 2014) or comparing models of fossil taxa to extant taxa with known behaviours (Püschel *et al*., 2018).

## VII. STABLE ISOTOPES

Stable isotopes are non‐radioactive forms of atoms with varying natural abundance, both geographically and in how they are preferentially incorporated into biomolecules (Koch, 2007; Clementz, 2012). Given these known trends in isotope abundance, we can reconstruct trophic structure, niche partitioning and habitat use from fossils (Frederickson, Engel & Cifelli, 2018).

Few stable isotope studies have incorporated troodontids. Enamel powder samples from seven dinosaur and mammal groups (Hadrosauridae, Ceratopsidae, Ankylosauria, Tyrannosauridae, Dromaeosauridae, Neoplagiaulacidae and Troodontidae) and one lizard (Varanoidea) were sourced from the Rainy Day site of the Late Cretaceous Oldman Formation of Canada (Cullen & Cousens, 2024). The targets in this study were stable strontium (^87^Sr/^86^Sr), carbon (δ^13^C), and oxygen (δ^18^O) isotope ratios as well as Sr/Ca and Ba/Ca elemental ratios. Overall, stable isotope and elemental ratios of troodontids fall between data of dromaeosaurids and ornithischians, straddling the ecological niche between primary and secondary consumers (Cullen & Cousens, 2024). Cullen & Cousens (2024) suggested troodontids at the site would be mixed‐feeding to plant‐dominant omnivores, differing from contemporary theropods.

The small range of the stable isotope ratios does not support the possibility that multiple troodontid taxa were measured in Cullen & Cousens (2024). The differences between individuals (*N* = 5) is more in line with intrageneric dietary variation (SD of δ^13^C: 2.45, δ^18^O: 2.52, ^87^Sr/^86^Sr: 0.00030 in Troodontidae) (Cullen & Cousens, 2024). The lack of variation within *Troodon* in this study could be attributed to its limited geographic scope, but, at least in this case, we consider shed *Troodon* teeth to be unlikely to represent multiple species.

## VIII. DISCUSSION

### (1) Troodontid diet

Little direct evidence for troodontid diet is known. *Jinfengopteryx elegans* provides one instance of plant consumption (Ji, 2005) in troodontids, which may or may not be typical for this taxon much less the clade as a whole. Thus, we are left with dietary proxies. Discussed at length in prior sections, we seek here to synthesise past findings into a more complete picture of troodontid diet. We generally support an omnivorous diet for *Troodon* and by extension late‐diverging troodontids.

Biogeochemical analysis (Cullen & Cousens, 2024) points directly to *Troodon* as an omnivore, with stable isotope and elemental ratios indicating a trophic niche between primary and secondary consumers. Omnivory is often used as a compromise in morphological studies when evidence for carnivory or herbivory is lacking, but this stable isotope and elemental evidence points to omnivory being a true signal rather than a compromise of noise. *Troodon*'s coarse denticles could help shear plant material (Holtz *et al*., 1998; Barrett, 2000) and aid in slicing through flesh (D'Amore, 2009). Nesting sites may have been frequented for opportunistic preying on hatchlings (Vickaryous *et al*., 1998) and to take advantage of abundant vegetation. The enlarged second digit claw may have helped trap animal prey (Oswald *et al*., 2023), aided in digging for fossorial prey animals (Simpson *et al*., 2010) and subterranean plant structures, and been useful in holding plant matter during feeding. In short, stable isotope and elemental ratios point to *Troodon* being an omnivore which in turn allows for the coexistence of otherwise contradictory interpretations of its anatomy and environment.

While the omnivorous palaeodiet reconstruction of *Troodon* may reasonably be extended to some other later‐diverging troodontids [e.g. *Linhevenator tani*, *Zanabazar junior* and *Saurornithoides mongoliensis* (Fig. 1)], diet for much of Troodontidae remains uncertain. Troodontids without denticles (e.g. *Mei long*, *Jinfengopteryx elegans*, *Byronosaurus jaffei*, *Xixiasaurus henanensis* and *Urbacodon itemirensis*) are perhaps the most obvious group that likely differed ecologically. The loss of denticles in a range of taxa from different periods and geographical locations (Fig. 1) implies a selective pressure to eliminate denticles. Possible driving factors for denticle loss include: taking advantage of different food sources; retaining a food source but changing to a hunting style that reduces reliance on disassembling prey (D'Amore, 2009) (e.g. swallowing food items whole); or a reduction in tooth turnover and associated energy costs (Section V.2). Ultimately, troodontids other than *Troodon* are understudied, which limits any attempts to address their palaeodiet. One major step forward would be stable isotope and elemental analysis of other troodontid‐bearing strata. An ideal candidate would be sections of the Yixian Formation which contain both *Mei long* and *Sinovenator changii* (Xu & Norell, 2004), which also allows for a comparison of troodontids with and without denticles.

As a whole, troodontids appear to have a uniquely strong anterior rostrum (Yun, 2025). As discussed above, this adaptation could equally serve as a tool to disassemble plant or animal matter, although we believe the prey‐shaking behaviour suggested by Yun (2025) requires additional evidence to be considered likely. At least some troodontids did consume a mixture of plant and animal tissue (Cullen & Cousens, 2024), and this rostrum specialised for manipulation likely served as a multifunctional tool akin to bird beaks (Friedman *et al*., 2019). The localised strength in troodontid jaws also calls into question whether past reports of their teeth requiring optimal loading to prevent failure (Torices *et al*., 2018) may only represent tooth function in the lateral jaw. Greater tooth strength in the anterior rostrum would allow for predation on restrained struggling prey (Oswald *et al*., 2023) and potentially even prey‐shaking behaviour (Yun, 2025). Alternatively, tooth failure may have exerted little selective pressure due to constant tooth shedding and regrowth (Vickaryous *et al*., 1998).

Troodontids were almost certainly less predatory than their close relatives the dromaeosaurids. Firstly, troodontid skulls are less robust than those of dromaeosaurids. They would not have had large jaw muscles, leading to a comparatively lower bite force than in dromaeosaurids (Pei & Xu, 2022), and troodontid jaws could not withstand as much dorsoventral bending as those of dromaeosaurids (Yun, 2025). Troodontids likely either swallowed prey whole or disassembled it with the relatively robust snout (Yun, 2025), limiting prey size. The relatively weak posterior jaw (Pei & Xu, 2022; Yun, 2025) would be unable to produce the wide‐gape (Tse *et al*., 2024) and saw‐motion biting (Sakamoto, 2010) hypothesised for dromaeosaurid predation. Secondly, troodontid hind limbs are less specialised for predation than in dromaeosaurids. While later‐diverging troodontids [e.g. *Linhevenator tani* and *Troodo*n] develop second digit claws which resemble those of dromaeosaurids (Xu *et al*., 2011) (Fig. 3), troodontid unguals were straighter and less curved than the ‘sickle claw’ of dromaeosaurids. In addition, troodontids had fewer pedal adaptations for grasping (Fowler *et al*., 2011) and more for cursorial movement (Gianechini *et al*., 2020; Ascari, 2021). While dromaeosaurids likely used their claws to restrain and kill large prey, troodontids were likely limited to immobilising small prey in the pes and killing with their jaws (Oswald *et al*., 2023).

So then, what role did troodontids play in their environments? *Troodon* has been suggested as ecologically analogous to coyotes (*Canis latrans*) (Cullen & Cousens, 2024). Coyotes will take food opportunistically from a wide variety of food sources, and as much as 40% of their diet may be plant matter (Watine & Giuliano, 2017). Extant ground‐dwelling birds (e.g. Galliformes, Struthioniformes) provide modern analogues and evidence for omnivorous paravians with herbivory‐dominated diets: lesser prairie chickens (*Tympanuchus pallidicinctus*) (Riley, Davis & Smith, 1993), red junglefowl (*Gallus gallus spadiceus*) (Arshad *et al*., 2000), and emus (*Dromaius novaehollandiae*) (Dunstan *et al*., 2013) include a large proportion of plant material (>50%) in their diets supplemented by a variety of invertebrates. We also highlight that this niche is common in small to medium carnivoran mammals: maned wolves (*Chrysocyon brachyurus*) (Massara *et al*., 2012), sun bears (*Helarctos malayanus*) (Sethy & Chauhan, 2018), African civets (*Civettictis civetta*) (Amiard, Kruger & Mullers, 2015), and European badgers (*Meles meles*) (Roper, 1994) all consume roughly equal levels of plant and animal matter. Across four families, species from carnivorous niches are able to infiltrate omnivorous lineages through key morphological adaptations while retaining many of their ancestrally carnivorous traits (Michaud *et al*., 2018). We propose *Troodon* and other coarse‐denticled troodontids represent analogous situations of infiltrating omnivorous niches from an ancestrally carnivorous niche. It remains to be seen if other troodontids were more carnivorous or if Troodontidae as a whole represents such a shift away from hypercarnivory, analogous to the divergence of Ursidae (Stirling & Derocher, 1990; van Heteren *et al*., 2016). One potential way to test these hypotheses is to examine variation in diet. In the analogue taxa above, diet is highly varied seasonally and regionally, e.g. African civets range from 25 to 75% plant matter in their diet depending on locality (Amiard *et al*., 2015). Intra‐tooth isotope variability has been used to identify seasonal diet shifts (Yang *et al*., 2024), although theropod teeth were replaced much more frequently than in mammals meaning at most two seasons may be captured in a given tooth (D'Emic *et al*., 2019). It is also unclear if this technique can be applied to Mesozoic fossils or if intra‐tooth isotopes would homogenise over these timescales.

### (2) Ancestral diet of Troodontidae

Theropods are believed to be ancestrally carnivorous (Nesbitt *et al*., 2006, 2009; Martinez *et al*., 2011), but later forms diverged ecologically and diversified. Herbivory, for example, has been found in maniraptorans (Kobayashi *et al*., 1999; Zanno, 2010; Zanno & Makovicky, 2011). It has been suggested that maniraptorans were ancestrally plant‐dominant omnivores, and hypercarnivory was secondarily derived in some paravian lineages (Zanno *et al*., 2009; Zanno & Makovicky, 2011). Within Deinonychosauria, it is generally agreed that dromaeosaurids were hypercarnivores (Fowler *et al*., 2011; Tse *et al*., 2024). The diet of troodontids is currently ambiguous, but at least some lineages were likely omnivorous (see Section VIII.1).

We reconstruct the hypothetical deinonychosaurian ancestor as more ecologically similar to troodontids than dromaeosaurids. Comparisons between dromaeosaurid and troodontid skulls suggests that a troodontid‐like gracile rostrum was the ancestral deinonychosaurian condition. Predatory traits (e.g. robust rostra, high bite force resistance) appear only in later‐diverging dromaeosaurids (Tse *et al*., 2024). The troodontid hind limb also resembles the ancestral paravian condition. Predatory grasping adaptations are developed only in the dromaeosaurid line (Fowler *et al*., 2011). Morphologically, the common ancestor to Deinonychosauria was likely much more similar to a troodontid than a dromaeosaurid. Although, rather than plant‐dominated omnivory (Cullen & Cousens, 2024), we consider animal‐dominated omnivory to be more likely in early‐diverging deinonychosaurians. Early‐diverging troodontids lack some herbivorous traits of later‐diverging taxa, like coarse denticles (Fig. 1) and densely packed dentition (Zanno & Makovicky, 2011).

Under this scenario, troodontids would be an evolutionary branch of early‐diverging deinonychosaurians that became more herbivorous [e.g. *Jinfengopteryx elegans* (Ji, 2005) and *Troodon* (Cullen & Cousens, 2024)] while dromaeosaurids increased specialisation to hypercarnivory. However, the above hypothesis of diet evolution remains tenuous due to uncertainties in the ecology of early‐diverging troodontids, as early‐diverging troodontids likely differ little from the ancestral deinonychosaurian. From largely herbivorous Maniraptoriformes [Zanno & Makovicky, (2011), but see Ma *et al*. (2020) regarding carnivory in Oviraptorosauria], a more carnivorous ancestral deinonychosaurian would align with established hypotheses of the paravian lineage as a whole veering towards carnivory (Zanno *et al*., 2009; Zanno & Makovicky, 2011). However, should continued research on the troodontid diet suggest a more herbivorous ancestral deinonychosaurian, the model would change. Rather than Paraves as a whole representing a shift towards carnivory and troodontids reversing back towards herbivory, carnivory would be convergently evolved in both the avialan and dromaeosaurid lineages. This would in turn beg questions of what drivers lead to this convergence; notably, Avialae and Dromaeosauridae are the only dinosaur lineages known to have developed flight (Pei *et al*., 2020).

### (3) Future directions

Current troodontid diet research is limited by focusing on isolated body parts, e.g. teeth or the hind limb. Feeding, however, is not an anatomically isolated action. It incorporates most of an animal's body working in concert (Montuelle & Kane, 2019). Due to the incomplete dietary information provided by past studies, we recommend other body parts which may help to build a more comprehensive view of the troodontid diet.

Since theropods do not chew (Zanno & Makovicky, 2011), the jaw and teeth are responsible for biting and securing food items. Manipulation and further disassembly of food is powered by the neck. There have been reconstructions of neck musculature in therizinosaurians, tyrannosaurids, allosaurids and ceratosaurids on the occipital region of the skull (Smith, 2015), providing better insight into the feeding motions of those theropod groups, and these configurations have been used to highlight differences in feeding style (Snively & Russell, 2007). The reconstruction of neck musculature can tell us how, or if, troodontids disassembled their food before swallowing. The fragmentary and incomplete state of many troodontid specimens makes it hard to map muscle insertions onto the cervical vertebrae, however, *Mei long* (IVPP V12733, DNHM D2514) and suspected *Philovenator* vertebrae (LH PV39) would be good study candidates. There is almost no literature on muscle reconstruction in paravian theropods except the extant bird work of Tsuihiji (2005, 2007), despite this study potential.

The forelimbs of troodontids have not been extensively studied in the context of food acquisition or consumption. Troodontid forelimbs significantly shorten over the course of evolution (Xu *et al*., 2011). *Linhevenator tani*, a late‐diverging troodontid, has one of the shortest humeri (relative to femur length) of any non‐avian theropod (Xu *et al*., 2011). *Troodon*, based on fragmentary fossil remains, appears to also have had greatly reduced forelimbs (Russell, 1969). Reduced arm length would seem to indicate a reduced role of forelimbs, but the size and form of the deltopectoral crest suggest the forelimbs were still functionally important (Lacovara, 2017). They may have allowed for activities such as digging or climbing (Xu *et al*., 2011), potentially reflecting a niche shift over time. A possible connection between reduction in forelimb size and herbivorous inclination has been discussed in oviraptorosaurians (Funston *et al*., 2020), revealing a potential area of study within Maniraptora. Troodontid forelimbs may have played an important role in food acquisition, which future work may reveal [e.g. on claws (Lautenschlager, 2014) and other areas].

**Fig. 6:**
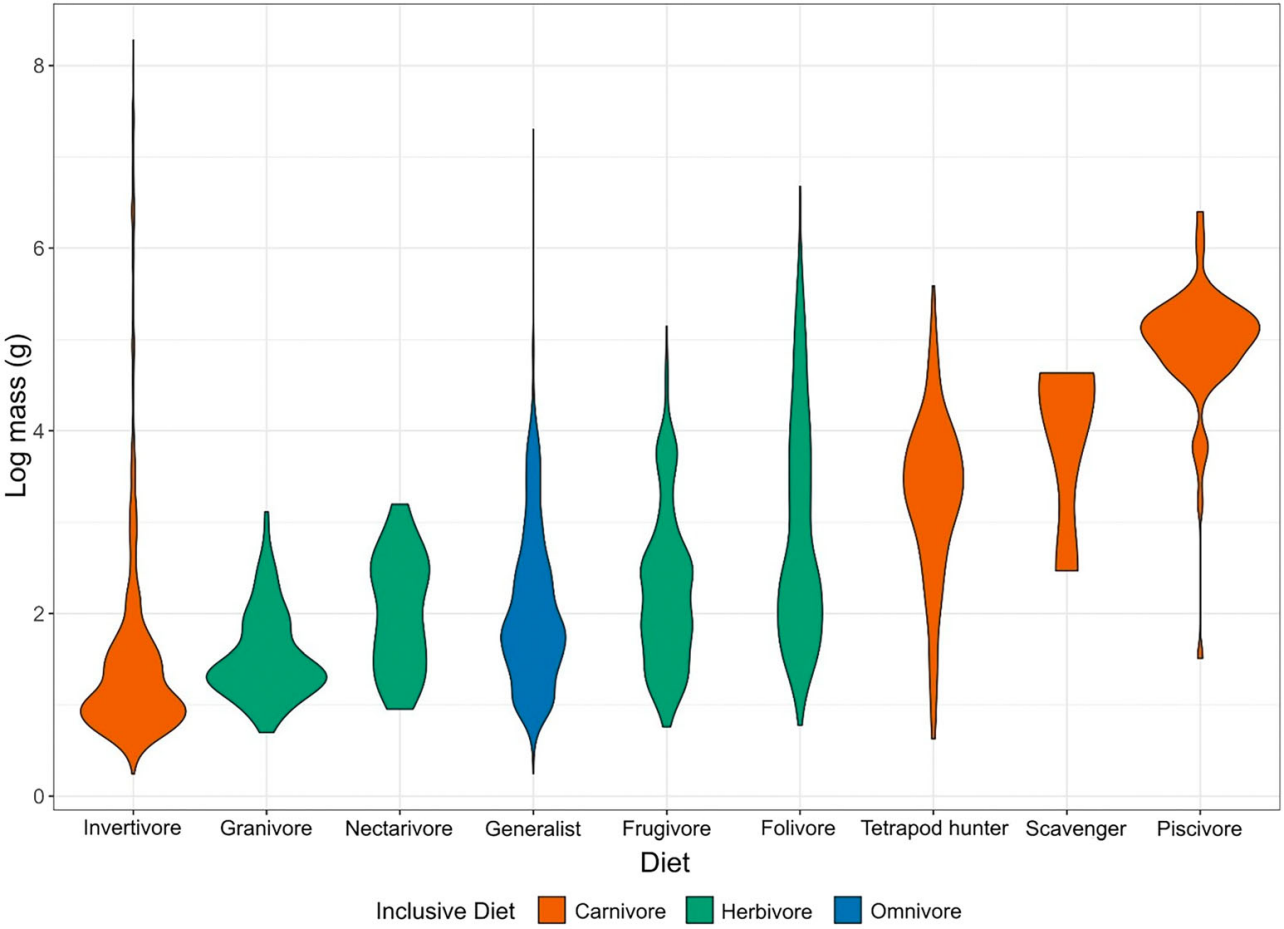
Violin plots of mammal mass, arranged by diet in order of ascending mean mass. Mass and diet data are taken from Wilman *et al*. (2014) (*N* = 5400). Diet categories were assigned with a simplified method based on Miller *et al*. (2022) and Miller *et al*. (2024). Any animal whose diet comprises more than 50% from one source was considered a specialist in that diet (e.g. *Felis catus* has a diet of 100% tetrapods, so is a tetrapod hunter) and any animal with no food source making up more than 50% of its diet was considered a generalist. Note diet categories are broader than in Miller *et al*. (2024). Possible factors to increase dietary resolution, e.g. separating terrestrial and aquatic mammals, are beyond the scope of this study.

The size of an animal provides a range of probable food items that it could have ingested. In extant birds, body mass has a major influence on feeding strategy (Bright *et al*., 2016; Pigot *et al*., 2020; Miller *et al*., 2024). Birds that take larger foods, whether plant or animal, tend to have larger body masses (Miller *et al*., 2022, 2024). Troodontids are generally small in size. *Mei long* was estimated to be approximately the size of a kiwi (*Apteryx* sp.), whereas *Troodon formosus* was estimated to have been approximately the size of a rhea (*Rhea americana*) (Sellés *et al*., 2021). However, mass and diet relationships in birds cannot be applied directly to troodontids. Troodontids are non‐volant, to current knowledge, and volancy places major restrictions on body size (Pei *et al*., 2020). Any examination of troodontid mass and diet should rely on non‐volant animals [e.g. ground‐dwelling avians (Dunstan *et al*., 2013) and mammals (Wilman *et al*., 2014)], although preliminary analysis (Miller *et al*., 2024) suggests mass is less constrained by diet in mammals than in birds (Fig. 6).

**Fig. 7:**
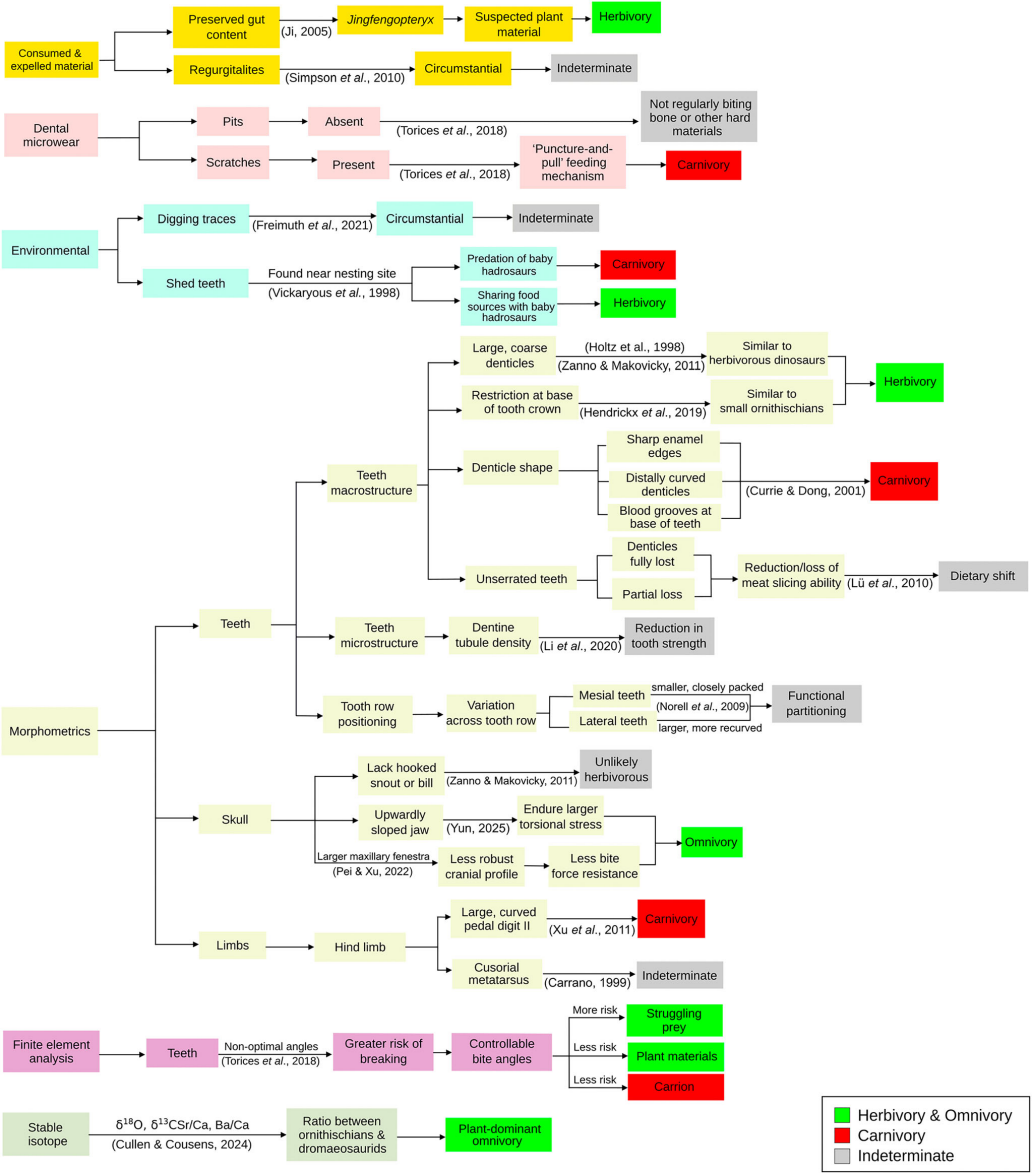
Summary of past research on troodontid diet, and their respective conclusions on dietary preferences. Overall, current evidence leans towards an omnivorous diet for Troodontidae.

Existing material on troodontids can provide substantial information on diet when approached from new angles. However, discovery of new material is still an essential part of pushing the boundaries of knowledge concerning extinct organisms. Figure 7 provides a summary of current dietary research on *Troodon*. Given the existence of other troodontids in North America (Zanno *et al*., 2011; Hartman *et al*., 2019), the diversity of North American troodontids is probably significantly underestimated at present. Machine learning has great potential to increase the speed of new material classification and readying it for further analysis. Machine learning's biggest advantage lies in its ability to automate repetitive, time‐consuming actions, which is compatible with the classification of large amounts of microfossils (Bahn, Alférez & Snyder, 2025). In the same vein, machine learning tools can also automatically sort microfossils from surrounding matrix (Kaye *et al*., 2015). Quick sorting, identification, and classification of vertebrate microfossils will improve the speed of troodontid specimen acquisition for further research. In the longer term, with more diet‐related data, we hope not only to build upon what we already know, but also to assess finer dietary differences that are currently out of reach, e.g. dietary differences linked to seasonality and latitudinal differences.

## IX. CONCLUSIONS

(1) Synthesising previous research on troodontid diet, *Troodon* was most likely a plant‐dominated omnivore. Outside of *Troodon*, the diet of other troodontid species remains under‐researched. The lack of denticles in certain troodontid taxa (e.g. *Byronosaurus jaffei*) likely indicates differences in food source or handling compared to troodontids with denticles. Increases in claw size and rostral strength over time likely also correlate with ecological change between early and late troodontids. Further information is still needed to create robust hypotheses for the diet of non‐*Troodon* troodontids.
(2) Deinonychosaurians and paravians appear to have been ancestrally omnivorous. We compile evidence to suggest that early troodontids provide a strong approximation for the ancestral ecology of Deinonychosauria. The ancestral diet of Deinonychosauria, in turn, can be combined with recent work on avialan diet for a fuller picture of paravian ecological evolution. Thus, to improve these ancestral dietary reconstructions, further study of early troodontids is essential.
(3) The small number of well‐preserved species limits what we currently know about troodontid morphology and ecology. The uncertain taxonomic status of *Troodon* further obscures potential ecological diversity in the clade. Troodontid fossils show morphological traits of both carnivores and herbivores, meaning the inclusion of non‐morphological lines of evidence is especially important when studying this clade. As the majority of palaeodiet studies have been carried out on *Troodon*, time may prove that other troodontid taxa will be less prone to these issues.
(4) This review shows that there is still much work to do to understand troodontid diet. Future discoveries of troodontid material could potentially clarify taxonomic confusion surrounding *Troodon*. In turn, this would provide a finer view of troodontid dietary niche partitioning in both North America and Asia, as well as a better understanding of the ancestral troodontid diet. Even without new specimens, there is plenty of under‐researched material to study in the meantime. Postcranial systems of troodontids, such as their forelimbs and neck, have the potential to contribute greatly to palaeodiet reconstructions. More comparisons with close relatives and modern ecological analogues also have the potential to push the field forward, whilst new fossils relevant to dietary reconstruction await discovery.

## AUTHOR CONTRIBUTIONS

M.P. conceived and designed the study. Y.C.F., C.V.M., and M.P read, analysed and synthesised the literature. Y.C.F. and M.P. wrote the first draft. Y.C.F. and M.P. produced the figures. Y.C.F., C.V.M., and M.P. contributed substantially to revisions. Y.C.F., C.V.M., and M.P. read and approved the final manuscript.

## Data Availability

All new data generated or analysed during the current study is in the manuscript itself.
